# Integrated Analysis of Microarray Data of Atherosclerotic Plaques: Modulation of the Ubiquitin-Proteasome System

**DOI:** 10.1371/journal.pone.0110288

**Published:** 2014-10-15

**Authors:** Zhe Wang, Dong Guo, Bin Yang, Jian Wang, Rong Wang, Xiaowei Wang, Qunye Zhang

**Affiliations:** 1 Key Laboratory of Cardiovascular Remodeling and Function Research Chinese Ministry of Education and Ministry of Public Health, Qilu Hospital, Shandong University, Jinan, Shandong, China; 2 Division of Endocrinology and Metabolism, Shandong Provincial Hospital affiliated to Shandong University, Jinan, Shandong, China; 3 Department of Neurology, Liaocheng People's Hospital, Medical School of Liaocheng, Taishan Medical University, Liaocheng, Shandong, China; 4 School of Information Science and Engineering, Zaozhuang University, Zaozhuang, Shandong, China; 5 School of Life Sciences, Shanghai University, Shanghai, China; Roswell Park Cancer Institute, United States of America

## Abstract

Atherosclerosis is a typical complex multi-factorial disease and many molecules at different levels and pathways were involved in its development. Some studies have investigated the dysregulation in atherosclerosis at mRNA, miRNA or DNA methylation level, respectively. However, to our knowledge, the studies that integrated these data and revealed the abnormal networks of atherosclerosis have not been reported. Using microarray technology, we analyzed the omics data in atherosclerosis at mRNA, miRNA and DNA methylation levels. Our results demonstrated that the global DNA methylation and expression of miRNA/mRNA were significantly decreased in atherosclerotic plaque than in normal vascular tissue. The interaction network constructed using the integrative data revealed many genes, cellular processes and signaling pathways which were widely considered to play crucial roles in atherosclerosis and also revealed some genes, miRNAs or signaling pathways which have not been investigated in atherosclerosis until now (e.g. miR-519d and SNTB2). Moreover, the overall protein ubiquitination in atherosclerotic plaque was significantly increased. The proteasome activity was increased early but decreased in advanced atherosclerosis. Our study revealed many classic and novel genes and miRNAs involved in atherosclerosis and indicated the effects of ubiquitin-proteasome system on atherosclerosis might be closely related to the course of atherosclerosis. However, the efficacy of proteasome inhibitors in the treatment of atherosclerosis still needs more research.

## Introduction

Atherosclerosis is the major cause of most of the serious cardio-cerebrovascular events and it accounts for approximately 30% of all deaths worldwide [1]. Although many studies have revolutionized our knowledge of the pathogenesis of atherosclerosis in the past several decades, we are still short of the full understanding of its mechanism and the ability to cure it. Nowadays, it is widely accepted that atherosclerosis is a typical complex multi-factorial disease with a long course and extremely complex pathological events [2]. Many genetic, epigenetic and environmental factors are closely related to the development of atherosclerosis.

Until now, lots of researches have shown that the deregulation of many molecules (such as DNA, miRNA and protein) in vascular endothelial cells (VEC), vascular smooth muscle cells (VSMC), monocyte-macrophage cells and other cells were involved in atherosclerosis [2], [3]. MiRNA is an important type of these molecules and they are a class of small noncoding RNAs (19∼25 nt) that can regulate the expression of its target genes. Each miRNA may regulate hundreds of mRNA targets and a single gene may be regulated by many miRNAs resulting in complex regulatory networks. Many studies have proven that miRNAs played critical roles in atherosclerotic processes, such as the VEC integrity, VSMC proliferation induced by ox-LDL and inflammatory response [4]–[7]. The abnormal DNA methylation is another common abnormality during the development of atherosclerosis. Some studies showed that the significant global DNA hypomethylation is considered one of the landmarks of advanced atherosclerosis and this abnormality affected the expression of many genes resulting in dysfunctions of a variety of cells (such as VEC, VSMC and immune cells) [8]. However, in spite of the hypomethylation of genomic DNA, the hypermethylation of many genes was also found. For instance, the DNA methylation in the promoter region of *FOXP3* in regulatory T cells was significantly higher in atherosclerosis than in normal vessel [9]. Moreover, there were intricate inter-regulations of miRNA and DNA methylation. For example, miR-29b could affect DNA methylation through targeting DNMT3b and epigenetically regulate the migration of human aortic smooth muscle cell (hASMC) [10]. Roles of miRNA and DNA methylation in atherosclerosis should be studied integratively. The recent development of many ‘omics’-scale technologies and their integration in the view of systems biology offered an opportunity to understand the complex interaction networks involved in atherosclerosis. Some studies have explored the mechanism of atherosclerosis using systems biology approach [11]–[14]. These studies were often focused on the characteristics of changes in atherosclerosis at a single level (such as mRNA, protein or DNA methylation). However, to our knowledge, the study which integrated the data of mRNA, miRNA and DNA methylation in atherosclerosis has not been reported.

Using microarray technology, we studied the global features of mRNA/miRNA expression and DNA methylation in atherosclerosis. Our results demonstrated that the global DNA methylation and expression of miRNA/mRNA were significantly decreased in atherosclerotic plaque than in normal vascular tissue. The integrated analysis of miRNA, mRNA and DNA methylation data revealed many genes and pathways that played crucial roles in atherosclerosis and also revealed some genes, miRNAs or pathways involved in atherosclerosis, but which have not been investigated until now. Many transcription factors were also significantly enriched in atherosclerosis. Moreover, our results showed that the proteasome concentration and overall protein ubiquitination in atherosclerosis were significantly increased and the proteasome activity was increased early but decreased in advanced atherosclerosis. These findings implied that the effects of ubiquitin-proteasome system (UPS) on atherosclerosis development might be closely related to the course of atherosclerosis. The efficacy of proteasome inhibitors in the treatment of atherosclerosis still needs more research.

## Materials and Methods

### Ethical Statement and tissue samples

The study in this paper has been approved by the Ethics Committee of Qilu Hospital and conformed to the Declaration of Helsinki. The written informed consents were obtained from either the patients or relatives. All coronary artery tissue samples were collected from the autopsy specimens that showed atherosclerotic plaques or normality in Qilu Hospital and Liaocheng People's Hospital.

### Nucleic acid extraction and microarray experiments

Total RNA, miRNAs and genomic DNA (gDNA) were isolated and treated as described previously from collected tissues samples [5], [15], [16]. The same quantity of purified total RNA/gDNA from 35 advanced atherosclerosis plaques or 35 normal coronary artery tissues (control) were respectively averaged into 7 atherosclerosis pools or 7 control pools. There were no significant differences between the same types of pools. Except for the presence of atherosclerotic plaques, other factors, such as age and gender, were not different between control sample pools and atherosclerosis pools. The RNA from 7 control pools and 7 plaque sample pools were labeled by Cy3 or Cy5 respectively and hybridized on human genome array chip V1.0 (Capitalbio, China). The treated gDNA from three control sample pools and seven plaque sample pools were hybridized with the methylation probes (cy5) and unmethylation probes (cy3) using GoldenGate Methylation Cancer Panel I (Illumina, CA). The qualified miRNA from 3 plaque sample pools and 3 control sample pools were labeled by Hy5 and the equal amount of miRNA taken from above six sample pools were combined and labeled by Hy3 as a common reference. Then the labeled miRNAs were hybridized on miRCURY LNA Array (v.14.0) (Exiqon, Denmark). After hybridization, slides were washed and data acquisition was done with a GenePix 4000B scanner (Molecular Devices, CA). The data of DNA methylation, mRNA/miRNA expression in our study have been submitted to the public data repositories, ArrayExpress. The accession numbers are E-MTAB-2458, E-MTAB-2459 and E-MTAB-2460.

### Differential and functional analysis of microarray data

The data of mRNA and miRNA microarray were filtered and normalized, and then the differentially expressed miRNAs/mRNAs (corrected p<0.05 and fold change ≥2) and differentially methylated sites (corrected P<0.05 and Δβ = |βAS-βctrl|≥0.3) in atherosclerosis were identified using GeneSpring GX 11.5 (Agilent Technologies, CA) and GenomeStudio Methylation Module v1.0. The Benjamini-Hochberg algorithm was used for controlling the false positive rate in multiple comparisons. The functional enrichment analysis and pathway activity simulation were conducted by IPA trial (Ingenuity Systems, CA) [17]–[19]. The region of −1500 bp - +200 bp to transcription start site (TSS) was considered to adequately cover the sequence that the promoters of most genes in eukaryotes might exist and the sequences in this region of differentially expressed and methylated genes were obtained from UCSC Genome Browser. The motif analysis of these DNA sequences was performed using DREME software. The transcription factors binding to the enriched motifs were analyzed and presented as Tagcloud. The details of the involved algorithms were described in Text S1.

### The construction and functional analysis of interaction network

The genes regulated at DNA methylation, transcriptional and post-transcriptional levels might have more significance in the development of atherosclerosis. So, these genes were selected as following. First, the genes that expression trends were opposite to those of miRNA were selected from the intersection of differentially expressed mRNAs and the potential target genes of the differentially expressed miRNAs. Then, these genes were merged with the differentially methylated genes. After that, the interaction network was constructed from the merged data by IPA based on the molecular interaction database including BIND v4.0, DIP v2.5, HPRD v9, INTACT v2.0 and BIOGRID v3.2.1 [17], [19]–[24]. The edges presented the direct interactions between the genes connected to each other and were non-directional. The core subnetworks in the interaction network were analyzed using KeyPathwayMiner that was a plugin of Cytoscape [25]. The details of the involved software and algorithms were described in Text S1.

### Quantitative real-time PCR (qPCR) and methylation-specific PCR (MSP)

The validation of differentially expressed mRNA was performed using SuperScript VILO Master Mix and TaqMan master mix according to the manufacturer's instructions in 7500 Real Time PCR System (Life Technologies, NY). The primers and probes of these genes were bought from Life Technologies. The human GAPDH was used as reference gene. The MSP of some differentially methylated sites were carried out as previously described [26]. All experiments were independently repeated 20 times.

### Assay of proteasome activity and concentration and Western Blot analysis

The proteasomes were purified from tissue samples using Proteasome Isolation Kit (Calbiochem, Germany). Then, the proteasome activity was measured by Proteasome-Glo assay kit (Promega, WI) following the manufacturer's instruction. The total proteins from tissues were prepared and the protein concentration was determined using a BCA protein assay. The concentration of 20S proteasome, the core subunit of proteasome, was measured as described in the manufacturer's manual using 20S Proteasome ELISA Kit (Creative Diagnostics, Shirley, NY). The amount of 20S proteasome was calculated per mg total protein. Additionally, aliquots of total proteins were used for Western Blot analysis with antibodies to TP53BP1, UCHL1, UBA3, UBE2S, PSMD1 and USP12 (Santa Cruz, CA). The β-actin was used as an internal control. All experiments were independently repeated five times.

### Statistical analysis

The data were expressed as means ±SEM or means ±SD. To determine the significance of differences between multiple groups, one-way ANOVA was used and groups were compared independently using post-hoc Student's unpaired t-test applying Bonferroni's correction. P<0.05 was considered statistically significant, with the exception of post-hoc comparisons, in which Bonferroni's correction was applied. All tests were two-sided. SPSS 15.0 (SPSS Inc., Chicago, IL) was used for all calculations.

## Results

### The characteristics of genome-wide DNA methylation in atherosclerosis

**Figure 1:**
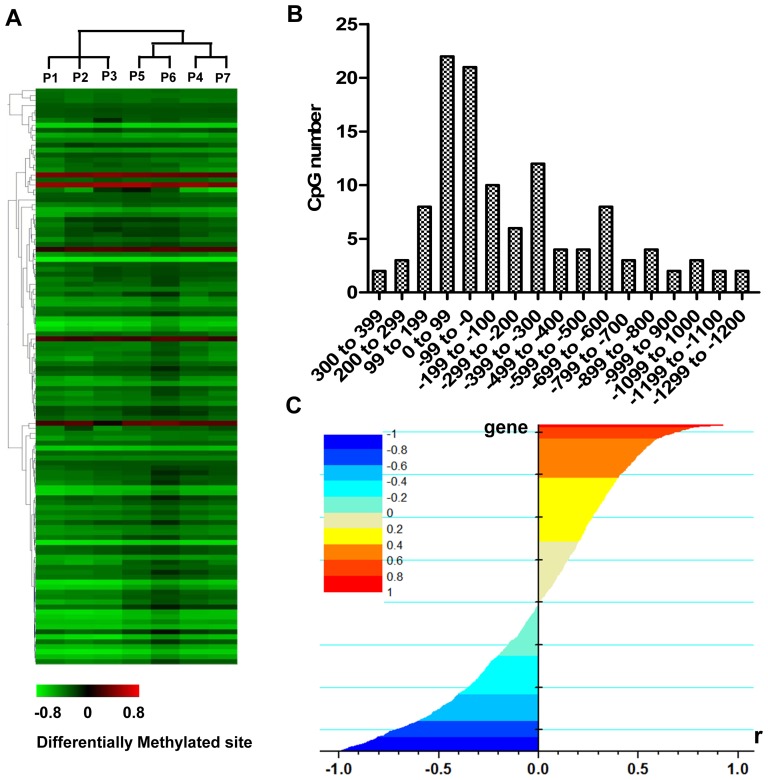
The characteristics of genome-wide DNA methylation in atherosclerosis. (**A**) The hierarchical cluster analysis of differentially methylated sites in atherosclerosis. The fold changes of DNA methylation compared to control sample pools were indicated by the color bar. P1–P7 indicated the 7 atherosclerosis sample pools. (**B**) Distribution analysis of the distance of differentially methylated sites to the transcription start site (TSS). The x-axis represented different distance ranges of differentially methylated sites to the corresponding TSS. (**C**) Pearson correlation coefficient of the fold changes of mRNA expression and the Δβ of corresponding DNA methylation. The x-axis represented the Pearson correlation coefficient (r). The color bar showed the different ranges of Pearson correlation coefficient.

The characteristics of tissue samples included in this study were shown in the Table 1. A part of these samples was used for mRNA, miRNA and DNA methylation microarray experiments as described in method. Age and gender did not differ significantly between the two groups. The analysis of DNA methylation microarray data showed that methylations of 97 genes or 116 GpG sites were significantly changed and the DNA methylation level was significantly lower in atherosclerotic plaques (AP) than in controls (Figure 1A). 52.6% of the differentially methylated sites were located in the region of −200 bp - +200 bp to TSS and this implied that the abnormal methylation of cis-acting elements might be primarily located in this region (Figure 1B). The analysis of correlation between DNA methylation and mRNA expression indicated that the mRNA expression levels of many genes were negatively correlated with their DNA methylation levels; however, there were still many genes, which were contrary to this. Namely, the DNA methylation of some genes did not inhibit their mRNA expression (Figure 1C). These results implied that the ultimate effect of DNA methylation was regulated by different mechanisms and was part of a complex regulatory network.

**Table 1:** Characteristics of the samples included in this study.

Group	EA	AA	Normal
**Female/male**	16/70	14/70	5/20
**age**	64±5	65±4	66±3
**tissue**	LAD	LAD	LAD
**Pathological type**	IV	V	normal
**death cause**	Accident	AMI	Accident
**area of occlusion**	37.25±14.24	81.08±4.64	0

**Note.** EA: early atherosclerosis; AA: advanced atherosclerosis.

LAD: left anterior descending; AMI: Acute myocardial infarction.

### Biological function analysis of miRNAs/mRNAs expression data

**Figure 2:**
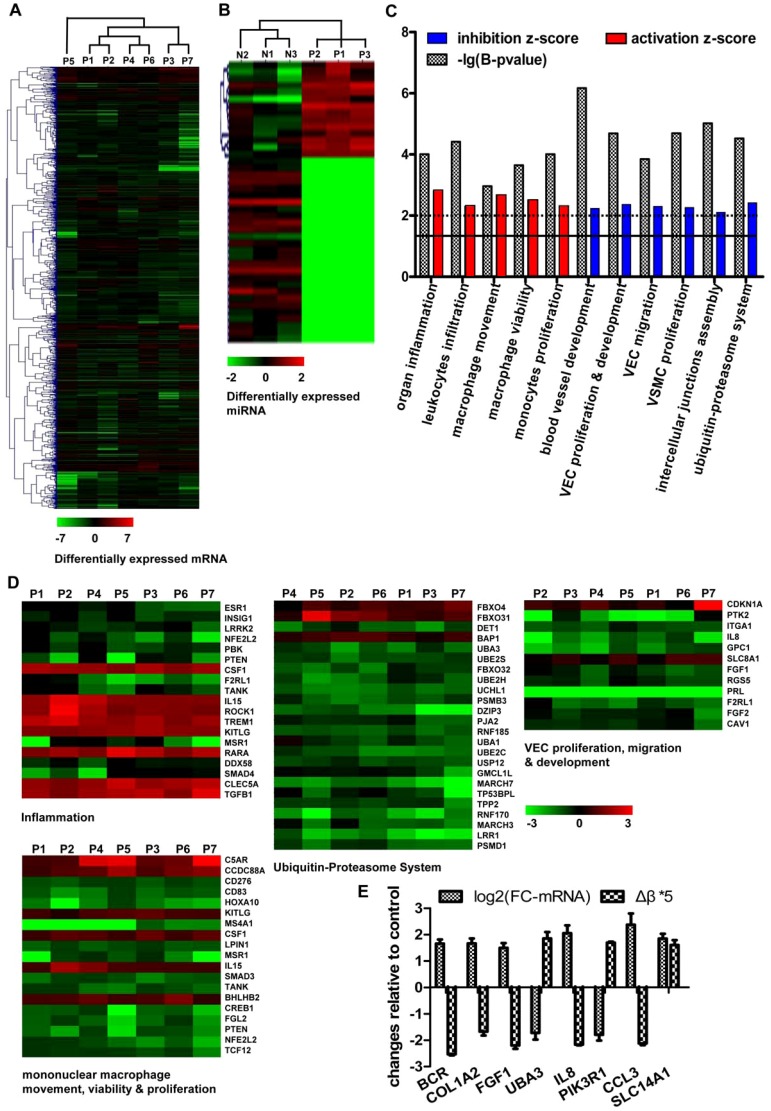
Expression characteristics and function enrichment analysis of differentially expressed miRNAs/mRNAs. (**A, B**) The hierarchical cluster analysis of differentially expressed mRNAs and miRNAs in atherosclerosis (P) compared to controls (N). The fold changes of expression compared to controls were indicated by the color bar. (**C**) The intersection of potential target genes of differentially expressed miRNAs and differentially expressed mRNAs was merged with the differentially methylated genes. Then the functional enrichment analysis and activation simulation of them was performed by IPA. B-pvalue indicated Bonferroni's corrected p value. The solid line showed the significant threshold of functional enrichment (>−lg(0.05) = 1.3). The dashed line showed the significant threshold of activation and inhibition simulation (|z|≥2). (**D**) The clusters of gene expressions of some enriched functions. The fold changes of expression were indicated by the color bar at the bottom. (**E**) Eight genes that were significantly regulated at DNA methylation and mRNA expression levels were confirmed by qPCR and MSP. The results of the t test showed that their expressions/methylations were significantly changed in atherosclerosis, P<0.05, Number of samples were 20.

The characteristics of the expression of mRNA and miRNA in atherosclerosis were analyzed using microarray technology as described in method 2. The results showed 975 genes and 41 miRNAs were significantly regulated and the global expression of mRNA and miRNA was decreased significantly in AP than in controls (Figure 2A, 2B). The clustering results showed that most of the expression profiles of mRNA/miRNA were consistent in the seven samples of AS. However, there were still differences in the expression features of some genes/miRNAs. The clustering of DNA methylation data had similar characteristics. It might imply that some molecules involved in the development of AS had individual differences. Additionally, 479 potential target genes of differentially expressed miRNAs were significantly regulated in atherosclerosis. These genes were merged with 96 differentially methylated genes and then the functional enrichment analysis of them was performed. The results showed that the processes of inflammation, leukocyte infiltration and macrophage migration were significantly enriched and activated; proliferation/migration of VEC and VSMC proliferation were also enriched but were markedly inhibited. One of the most significantly suppressed functions was the ubiquitin-proteasome pathway and the Z-score of activity simulation of this pathway was −2.42. The inhibition of UPS could result in the disturbance of normal protein turnover and more serious abnormality of many pathways. Moreover, the function of intercellular junctions assembly were also significantly enriched and inhibited and this might lead to further enhance the permeability of the endothelium and the accumulation of many pro-atherosclerotic factors (such as ox-LDL and proinflammatory cells) in subendothelium resulting in the development of atherosclerosis (Figure 2C, 2D). Eight genes were significantly regulated at DNA methylation and mRNA expression levels in atherosclerosis. These genes were involved in inflammation, collagen synthesis and PI3K/AKT pathways and might play important roles in the atherosclerosis process. The expression and methylation of them were validated using qPCR and MSP (Figure 2E).

### Interaction network analysis of the integrated data

**Figure 3:**
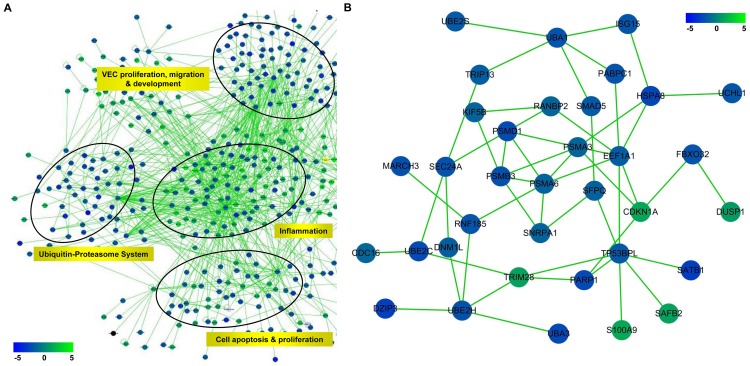
Interaction network based on integrated data at different levels in atherosclerosis. (**A**) The part of the constructed interaction network involved in atherosclerosis. It was shown that the functions of UPS, VEC proliferation/migration/development, Inflammation and cell apoptosis/proliferation were enriched significantly. The fold changes of expression of nodes (genes) were indicated by the color bar in the lower left corner. (**B**) The sub-network in which UPS was enriched was shown and the genes associated with UPS were mainly inhibited. The fold changes of expression of nodes (genes) were indicated by the color bar in the upper right corner.

The interaction network was established based on the interaction information from various interaction databases and the integrated data of differentially expressed miRNAs/mRNAs and methylated genes in atherosclerosis. The whole network was constructed by more than 6000 nodes and 10,500 edges. Then, the function enrichment analysis of this network was performed and some sub-networks in which certain function was relatively enriched were found. Figure 3A showed a part of the whole network and indicated the functions of UPS, VEC proliferation/migration/development, Inflammation and cell apoptosis/proliferation were enriched significantly (Network S1). The sub-network in which UPS was enriched was shown in figure 3B and the genes associated with UPS were mainly inhibited (Network S2). The results indicated that UPS might be markedly regulated and play important roles in the process of atherosclerosis.

### Motif analysis and the enriched transcription factors

**Figure 4:**
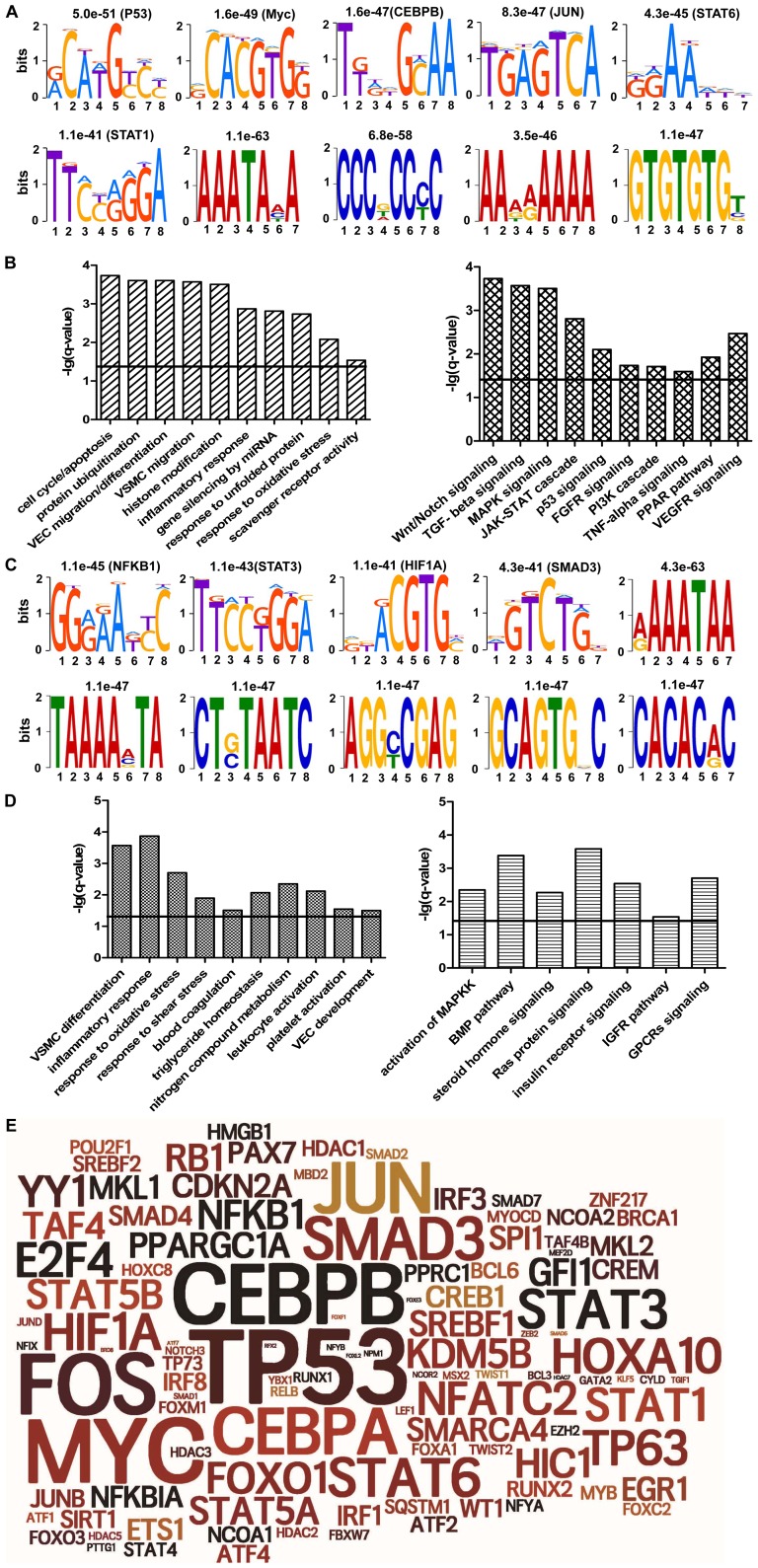
Motif analysis and functional enrichment of transcription factors. (**A, C**) The motif analysis of promoter sequences of 975 differentially expressed genes (A) and 97 differentially methylated genes (C) were performed using DREME software. The top ten significantly enriched motifs were shown and the E-value and corresponding TFs was labeled on the top of each motif respectively. (**B, D**) The pathway and functional enrichment analysis of the enriched motifs shown in A (B) and shown in C (D) were performed using IPA. The enriched functions (pathways) were shown in the left (right) panel. The solid line showed the significant threshold of enrichment analysis (>−lg(0.05)  = 1.3). (**E**) The presentation of enriched transcription factors related to the differentially expressed/methylated genes in style of Tagcloud. The bigger the font of the transcription factor, the more differentially expressed/methylated genes regulated by it.

Many transcription factors (TFs) have relatively specific DNA binding sequences, namely motif. Motif analysis of differentially expressed genes would be helpful for revealing the TFs that led to abnormal expression of genes. So, promoter sequences of 975 differentially expressed genes were analyzed and the top ten enriched motifs were shown in figure 4A. There were some binding motifs of TFs including P53, Myc, CEBPB, JUN and STAT6/1. This result suggested that these TFs might play vital roles in the aberrant expression of genes in atherosclerosis. Furthermore, the function/pathway analysis of the enriched motifs and TFs showed that the enriched functions/pathways included protein ubiquitination, endothelial cell function, cell cycle/apoptosis and inflammation as well as Wnt, SMAD and TGF-beta signaling pathways (Figure 4B). Moreover, the functions related to histone modifications and gene silencing by miRNA were also significantly enriched. This result implied that epigenetic regulation also played key roles in atherosclerosis. In addition, promoter sequences of 97 differentially methylated genes were analyzed and the top ten enriched motif were showed in figure 4C. There were also some binding motifs of TFs including SMAD3, STAT3, HIF1A and NFKB1. Most of the enriched functions and pathways were similar to those of the result of differentially expressed genes (such as apoptosis, VEC migration/differentiation, TNF-α, and ubiquitination pathway). However, some functions and pathways were more significantly enriched (such as VSMC differentiation, response to laminar fluid shear stress, triglyceride homeostasis and insulin receptor signaling) (Figure 4D). Then, we presented the TFs revealed by motif analysis of differentially expressed/methylated genes by Tagcloud. As shown in figure 4E, the bigger the font of the TF, the more differentially expressed/methylated genes regulated by it. The result clearly showed that these TFs including TP53, FOS, CEBPB/A, HIF1A and NFKB1 played important roles in atherosclerosis.

### Inhibition of activity of UPS in advanced atherosclerosis

**Figure 5:**
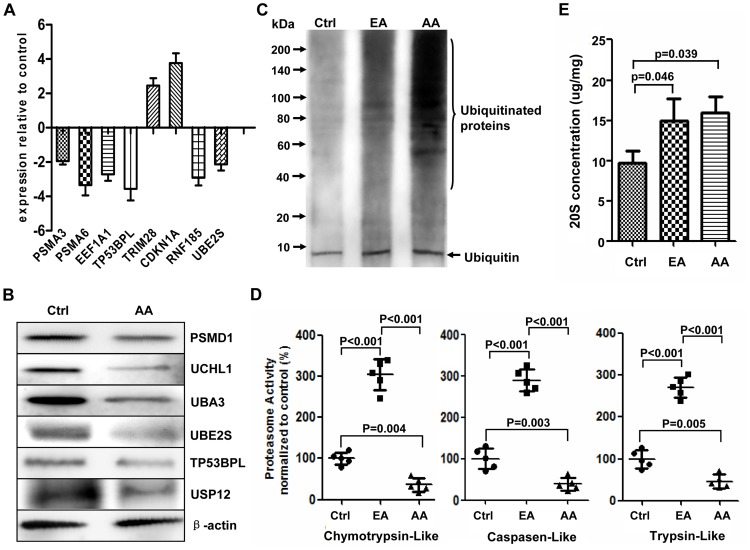
Analysis of the ubiquitin proteasome system in atherosclerotic plaques. (**A**) Eight key genes in figure 3B were validated by TaqMan qPCR in 20 atherosclerotic plaques and 20 controls (n = 20). The results of the t test indicated they were all significantly modulated in advanced atherosclerotic plaques compared to the normal controls (p<0.05). The positive fold change indicated upregulation and negative fold change indicated downregulation. (**B**) The representative results of the Western Blot of some key genes in UPS in advanced atherosclerosis (AA) and normal controls (Ctrl), n = 5. (**C**) The representative Western Blot of the overall protein ubiquitination in Ctrl, early atherosclerosis (EA) and AA, n = 5. (**D**) The proteasome activity (chymotrypsin-like, trypsin-like and caspase-like activity) in Ctrl, EA and AA was assayed. The three kinds of protease activities of proteasome in EA and AA groups were all normalized to the corresponding activity in Ctrl group. n = 5, P values were shown in the figure. (**E**) The concentration of 20S proteasome (the core subunit of proteasome) in Ctrl, EA and AA was determined. Its amount was calculated per mg total protein. P values were shown in the figure. n = 5. There was no significant difference between EA and AA.

The integrated analysis of the data at different levels including mRNA, miRNA and DNA methylation suggested that many genes of the UPS, such as E1, E2 and E3 enzymes, deubiquitinating enzymes and proteasome proteases were significantly down-regulated in atherosclerosis (Figure 2D). These results demonstrated that the activity of UPS was markedly inhibited in advanced atherosclerosis (the area of occlusion >75%). To confirm this conclusion, some key genes in UPS regulated in atherosclerosis were verified by TaqMan qPCR and Western Blot respectively and the results showed that the expressions of selected genes were all significantly regulated (Figure 5A, 5B). Moreover, our results indicated that the activity and concentration of proteasome as well as overall protein ubiquitination were significantly increased in early atherosclerosis (the area of occlusion <25%) than in normal vessel walls of coronary artery. However, although the overall protein ubiquitination and proteasome concentration were also significantly increased, the proteasome activity was markedly decreased in advanced atherosclerosis than in early atherosclerosis and control (Figure 5C–5E). The decreased activity of proteasome might cause the accumulation of ubiquitinated proteins resulting in the increase of the overall levels of protein ubiquitination. These results implied that the UPS might play different roles in different stages of atherosclerosis development and should be studied in more details. It should be noted that the samples in the assay of proteasome concentration and activity were relatively few in this study. Therefore, although the difference between groups was significant (p<0.05), the false positives caused by sampling, individual differences and the experimental errors should be paid more attention.

## Discussion

Atherosclerosis is a complex multifactorial disease. Although many theories of the pathogenesis of atherosclerosis have been proposed, so far none of these theories can explain all the features of atherosclerosis [1]–[3]. The complexity of atherosclerosis determined that the integrated analysis of data at multiple levels would be helpful to reveal the unique characteristics of this intricate disease more realistically. However, until now there is still no consummate approach for integrating and analyzing the heterogeneous data, such as DNA, RNA and proteins data. In our study, the omic data at different levels were integrated by combining the intersection of differentially expressed mRNA and the potential target genes of the differentially expressed miRNAs. Then, these genes were merged with the differentially methylated data. The networks were constructed based on interaction databases using these integrated data. This strategy was relatively simple and to a certain extent, reflected advantages of the systems biology approach. It might be a practical strategy used in the systems biology study of complex diseases under the current technical conditions.

Traditionally, DNA methylation has been considered as leading to gene silencing. However, our study showed that the levels of DNA methylation of many genes were increased, while their expressions were also upregulated. It seemed that the DNA methylation did not inhibit the expression of genes. There were many reasons for the result. For example, recent studies have demonstrated that the effects of DNA methylation on gene expression were related to the position of CpG sites in the gene. There were negative methylation-expression correlations for CpG located near a gene's TSS and positive correlations for CpG located in its body [27]. Additionally, DNA methylation might effect on genetic variation that could lead to the increased gene expression [28]. Moreover, histone modifications could regulate gene expression through modulating chromatin structure, which might alter the regulatory effect of DNA methylation on gene expression [29]. In fact, many studies in recent years have shown a more complex picture of the effects of DNA methylation on gene expression, and the relationship between DNA methylation and gene expression remains unclear [27]–[32].

Our results showed many abnormalities of crucial functions and pathways in the pathogenesis of atherosclerosis, for instance, increased inflammation, platelet activation, and functional inhibition of VEC as well as pathway of TGF-β, JAK-STAT, PI3K/AKT and TNF-α. Some genes involved in these pathways or functions were not significantly regulated at DNA methylation, mRNA or miRNA level. The sole analysis of the data at different levels without using the integrative systems biology approach would undoubtedly lose the valuable information. The sequence motif is a nucleotide or amino acid sequence pattern that is widespread and has a biological significance, such as binding to transcription factors. The sequence motif analysis could reveal the biological significance inherent in the data at different levels from the structural point of view. Our study unveiled enrichment of motifs specific to some TFs and indicated that these TFs might play important roles in the abnormal gene expression in atherosclerosis. Some of them (such as NFKB1, P53, Myc, and HIF1A) had been reported to be key regulators of inflammation, VSMC/VEC function and many other biological processes in atherosclerosis [33]–[36].

Furthermore, we constructed the interaction network from the integrative data of mRNA, miRNA and DNA methylation and revealed core subnetworks and nodes. Many of them had been reported to play important roles in atherosclerosis. For example, MiR-33b could regulate fatty acid metabolism and inhibition of miR-33a/b promoted the regression of atherosclerosis. Inhibition of SYK or CDKN1A might prevent against atherosclerosis development [37]–[39]. However, some of these nodes had not been reported in the study of atherosclerosis until now. For example, MiR-519d had oncogenic roles in hepatocellular carcinoma and it targeted CDKN1A/p21, PTEN and TIMP2, which had important roles in atherosclerosis. Overexpression of miR-519d was associated with human obesity [40], [41]. Moreover, SNTB2 might be involved in the regulation of insulin secretion [42]. These genes or miRNAs were likely to play important roles in atherosclerosis and were worth further study.

UPS is the main approach that degrades misfolded or damaged proteins and closely related to oxidative stress and unfolded protein response. UPS played important roles in atherosclerosis, but there was still controversy about its regulation, roles and therapeutic value. It was reported that proteasome activity and ubiquitinated proteins were significantly upregulated in atherosclerosis and the activation of UPS promoted the development of atherosclerosis [43], [44]. UPS inhibitors could markedly reduce neointima formation in balloon injured carotid arteries and slow the development of atherosclerosis [45], [46]. However, other studies showed that UPS prevented pigs from atherosclerotic lesion formation in coronary arteries and the inhibition of UPS worsened atherosclerosis [47], [48]. Our results showed that ubiquitinated proteins were significantly increased, but the UPS activity was significantly inhibited in advanced atherosclerosis. All these contradictory findings made it tempting to speculate that the ultimate roles of UPS in atherosclerosis depend on multiple factors, such as the stage of atherosclerosis, cell type and dose of proteasome inhibitors [49]. In our opinion, in the initial stage of atherosclerosis, the oxidized/misfolded proteins were increased by a variety of harmful stimuli and then the UPS was activated, resulting in the enhanced degradation of damaged proteins. But in advanced atherosclerosis, the long-term stimulation of harmful stimuli inhibited the activity of UPS and led to the accumulation of damaged proteins and severe abnormality of various cell functions, thus promoting the development of atherosclerosis. Nevertheless, in order to definitely answer the question whether an increase or a decrease in UPS activity promotes or blocks the atherosclerosis progression, it is necessary to perform more in-depth studies evaluating the effect of the selectively modulation of the UPS in different stages of atherosclerosis.

In conclusion, using a relatively simple strategy, we found many mRNAs/miRNAs, DNA methylations and signaling pathways that were widely considered to play crucial roles in atherosclerosis (such as activated inflammatory response, impaired endothelial function, NFKB, TGF-β and PI3K pathway). Our study also revealed some genes, miRNAs (e.g. miR-519d and SNTB2) or pathways that have not been fully investigated in atherosclerosis until now. Moreover, the roles and therapeutic value of UPS in atherosclerosis were worthy of more thorough investigation. It should be pointed out that our study focused on revealing the whole view of features and network in atherosclerosis, and did not further investigate them in different cell types of atherosclerosis. In the following study, we will solve this limitation and reveal in more detail the regulatory networks involved in atherosclerosis.

## Supporting Information

Network S1 **The xml file of the network presented in** **figure 3A** **.**
(XML)

Network S2 **The xml file of the network presented in** **figure 3B** **.**
(XML)

Text S1 **Microarray data analysis and networks construction.**
(DOC)
