# Supplementary material for: Integrated Analysis of Microarray Data of Atherosclerotic Plaques: Modulation of the Ubiquitin-Proteasome System
Source: PLoS One. 2014 Oct 15;9(10):e110288. doi: 10.1371/journal.pone.0110288 (PMC4201353; doi:10.1371/journal.pone.0110288)
Supplement: Text S1 — Microarray data analysis and networks construction. (DOC) [file pone.0110288.s003.doc]

**Microarray data analysis and networks construction**

After the acquisition of raw data of miRNA and mRNA microarray, RMA/GCRMA algorithm was used to correct background，normalize data，calculate expression values at probe level and summarize probes. SAM algorithm was used to analyze differentially expressed genes/miRNAs. The above algorithms were incorporated into GeneSpring GX v11.5. In gene expression analysis, most normalization algorithms are performed under the assumption that the majority of genes are not differentially expressed. However, this assumption is not correct for the analysis of DNA methylation microarray data. So, the background normalization algorithm was used to analyze the DNA methylation microarray data. The algorithm is simple and does not rely on the assumption that the majority of genes are not differentially expressed. In this manuscript, the beta value (ß) which is the ratio of intensities between methylated and unmethylated alleles was used to estimate the methylation level of the CpG locus. Its formula is as follows: β=*Max*(*Cy*5,0)/[*Max*(*Cy*3,0)+*Max*(*Cy*5,0)+100]. The t test with multiple comparison correction algorithms was used to analyze the regulation of DNA methylation. The above algorithms were all incorporated into GenomeStudio Methylation Module v1.0.

IPA (Ingenuity Pathway Analysis, Ingenuity Systems) was used for functional enrichment and detection of significant pathways. The principal algorithm/method embedded in IPA for biological functions/pathway enrichment was mainly singular enrichment analysis (SEA). Briefly, they searched through the list of selected genes and determined genes that were involved in the respective Knowledge Base (including GO term, canonical signal transduction or metabolic pathways). Then, Fisher's Exact algorithm was used to calculate the probability of which each functional gene set was enrichment. Only the biological functions/pathways (Bonferroni's corrected p-value<0.05) were considered significant enrichment. The activation status of the functions/pathways were predicted using IPA Upstream Regulator Analysis Tool by calculating a regulation Z-score and an overlap p-value, which were based on the number of known target genes of interest pathway/function, expression changes of these target genes and their agreement with literature findings. It was considered significantly activated (or inhibited) with an overlap p-value≤0.05 and an IPA activation Z-score≥2.0 (or ≤−2.0). The detailed descriptions of IPA analysis are available under “Upstream Regulator Analysis”, “Biological Functions Analysis”, and “Ingenuity Canonical Pathways Analysis” on the IPA website (http://www.ingenuity.com).

In this study, IPA was used to analyze the integrated data of mRNA/miRNA expression and DNA methylation and create the interaction networks for functional analysis based on the Ingenuity Knowledge Base. The Ingenuity Knowledge Base is proprietary database of IPA that is frequently updated and manual corrected. The interaction information of the Ingenuity Knowledge Base comes from peer-reviewed journals and both public and private biomedical databases, including the databases described in the manuscript. The redundant/duplicate interaction information from all above resources was deleted. The redundant/duplicate names in the submitted gene list were also deleted. The rules of deletion are mainly based on Ingenuity Ontology established by IPA Company. Ingenuity Ontology is an Ontology database that is manually reviewed by experts and contains a variety of synonyms and homonyms to ensure semantic consistency. It is convenient to remove the redundancy and ensure the identity of the target using Ingenuity Ontology. In order to construct networks, each identifier in the submitted gene list that the redundant/duplicate names had been deleted was mapped to its corresponding gene object in the Ingenuity Knowledge Base. These genes, called focus genes, were overlaid onto a global molecular network in the Ingenuity Knowledge Base. Networks of these focus genes were then algorithmically generated based on their connectivity. The involved algorithm was based on a multi-stage, heuristic algorithm that executes six steps to try to satisfy all of the above goals. The IPA network generation algorithm iteratively constructs networks that greedily optimize for both interconnectivity and number of focus genes under the constraint of a maximal network size. The details and technical requirements of the algorithms in each step are available in IPA website (http://www.ingenuity.com/wp-content/themes/ingenuitytheme/pdf/ipa/IPA-netgen-algorithm-whitepaper.pdf) [17]. The edges between the nodes (genes) of the interaction networks were non-directional and only indicated the interaction between the nodes (genes).

To find the core subnetworks of the whole network, we first used KeyPathwayMiner, a plugin of Cytoscape, to analyze the whole interaction network constructed by IPA. KeyPathwayMiner could efficiently extract all maximal connected sub-networks. These sub-networks contained the genes that are mainly regulated in most cases studied. Two slightly varying models (INES and GLONE) felled into the class of NP-Hard optimization problems. To tackle the combinatorial explosion of the search space, a set of exact and heuristic algorithms was designed in this software. The details of the algorithm could be found in reference 25. Then, functional enrichment of genes involved in the extracted sub-network was performed. The sub-networks with significant functional enrichment (such as UPS and inflammation, etc) were considered as the core sub-networks that had functional significance in the development of AS and the following studies were focused on them. In order to show more clearly, the cytoscape was used to present sub-networks with the edge-weighted spring-embedded layout algorithm.
